# Rwandan nursing students’ knowledge, attitudes and application of evidence-based practice

**DOI:** 10.4102/curationis.v43i1.2005

**Published:** 2020-01-28

**Authors:** Favorite Iradukunda, Pat M. Mayers

**Affiliations:** 1 Faculty of Health Sciences, University of Cape Town, Cape Town, South Africa; 2 Division of Nursing and Midwifery, Department of Health and Rehabilitation Sciences, University of Cape Town, Cape Town, South Africa

**Keywords:** nursing education, evidence-based practice, survey, Rwanda, knowledge, attitudes and application

## Abstract

**Background:**

Evidence-based practice (EBP) plays a key role in improving health outcomes of a country’s population; however, the teaching of EBP is often theoretical and inconsistent, resulting in negative attitudes and limited application.

**Objectives:**

The aim of this study was to determine the knowledge, attitudes and application of EBP by nursing students at a school of nursing in Rwanda.

**Method:**

A total of 82 third- and fourth-year nursing students completed the survey. Univariate statistical analyses were performed to explore the distribution of data. Chi-square tests were utilised to examine the relation between knowledge, attitudes and application of EBP with the year of study.

**Results:**

Most students reported being knowledgeable of the steps of EBP, with a range of 84% – 92%. However, 50% reported negative attitudes and only 12% reported daily searches for evidence. The main reported barriers to the application of EBP were lack of knowledge, lack of time and lack of examples or role models.

**Conclusion:**

Knowledge about EBP does not necessarily positively influence student attitudes. Evidence-based practice should be integrated into the theoretical and practical component of the nursing curriculum to promote the effective application of EBP by nursing students.

## Introduction and background

The term ‘evidence-based practice’ (EBP) refers to a combination of three interconnected concepts: clinical experience, research findings and patient values or preferences (Straus et al. 2011). Evidence-based practice is essential for improving patient health outcomes (Craig & Smyth 2012). As a core competency for health professionals, it is important that students are taught how to search and apply evidence from the early stages of their academic training (Llasus, Angosta, & Clark 2014).

In low- and middle-income countries, numerous barriers to high-quality performance and effective implementation of EBP have been reported (Jordan, Bowers & Morton 2016; Khammarnia et al. 2015). Shayan, Kiwanuka and Nakaye (2019) have categorised these as institutional-, interdisciplinary- and nurse-related barriers. Limited integration of EBP in education and clinical practice is often observed in African countries owing to the longstanding shortage of human or material resources (Forland et al. 2013). Incorporation of EBP into the culture of an organisation is a long process requiring leadership and resources (Patelarou et al. 2017).

Rwanda is one of the low-income countries in the world; however, through determined governmental implementation of policies, health care in Rwanda has markedly improved over the last 25 years (Abbott, Sapsford & Binagwaho 2017; Binagwaho et al. 2014; Sayinzoga & Bijlmakers 2016). The Rwandan health care system aims to be patient-centred and evidence-based (Moen et al. 2015; Rusatira et al. 2016), which requires ongoing improvement of the quality of health care professional education. Nurses need to be prepared for EBP, and the most effective and efficient means to ensure this is to provide learning opportunities for EBP in the nursing curricula (McInerney & Suleman 2010; Melender, Mattila & Häggman-Laitila 2016). Nurses have and continue to play a major role in this improvement. It is therefore important to ensure that nurses are prepared to meet the current health care needs using procedures that are evidence-based. This study aimed to determine the knowledge, attitudes and application of EBP by nursing students at a nursing school in Rwanda, and to describe the barriers to the implementation of EBP.

## Literature review

Successful application of EBP requires knowledge and skills to develop appropriate research questions, search and critically appraise relevant literature and evaluate the transferability of research evidence into clinical practice (Craig & Smyth 2012; McInerney & Suleman 2010; Straus et al. 2011). Lack of knowledge of EBP steps and principles may lead to negative attitudes towards EBP and result in their limited application or no application at all (Melnyk et al. 2012; Saunders & Vehviläinen-Julkunen 2016). This lack of knowledge is often associated with limited training in research and EBP (Sim, Jang & Kim 2016).

In addition to knowledge, positive attitudes are necessary for a successful application of EBP. Attitudes towards EBP tend to vary with knowledge and skills (Stokke et al. 2014). A descriptive survey of 20 000 nurses across the United States regarding the state of EBP reported negative attitudes towards EBP because of being overwhelmed by the volume of information they had to navigate to obtain accurate evidence (Melnyk et al. 2012). A lack of critical appraisal and advanced literature search skills may contribute to the negative attitudes towards EBP (Tacia et al. 2015), which may be minimised by appropriate teaching and practice of these skills (Melnyk et al. 2012).

There is evidence that knowledge, attitudes and application of EBP may be associated with the level of nursing education and integration of EBP in nursing education curricula. Mashiach Eizenberg (2011) reported that nurses who graduated from a degree programme that included EBP were more likely to apply EBP than those without a degree. Melnyk et al. (2012) identified a correlation between the level of education and the clarity about the steps of EBP; nurses with higher levels of education were clearer about EBP steps (Melnyk et al. 2012). In a quasi-experimental study conducted in Korea to evaluate the effect of education programmes on EBP implementation, nurses reported an improvement in their critical appraisal of research, searching skills and the ability to utilise and discuss research findings as a result of the integration of an EBP programme in their training (Sim et al. 2016). Integration of EBP in academic curricula and clinical training has been recommended (Melnyk et al. 2014). Such integration in the early stages of nurse training facilitates the development of nursing graduates with positive attitudes towards research and EBP, which, in turn, leads to rigorous application of EBP in clinical practice. It is the role of the universities, the nursing leadership and the policy-makers to ensure that nursing students and nurses are adequately equipped with EBP knowledge and skills (McInerney & Suleman 2010; Melnyk et al. 2012).

Effective integration of EBP in nursing curricula should include theoretical and practical (clinical) components. Theoretical teaching of EBP prepares students to be able to search and appraise the literature, while clinical teaching enables them to apply EBP into practice (Balakas & Sparks 2010; Finotto et al. 2013). In a review of strategies for teaching EBP in nursing education, various learning methods and opportunities were identified, including problem-based learning, interactive and clinically integrated teaching strategies, participation in research projects, conducting small research projects and journal clubs (Horntvedt et al. 2018). Online learning modules have also been reported to be useful (Patelarou et al. 2017). Simulation provides further EBP learning opportunities, as simulation scenarios can be developed from the existing evidence-based guidelines; this allows students to practise EBP principles in a safer environment (Waxman 2010).

In most nursing curricula into which EBP has been integrated, the integration is based on the five steps of EBP, commonly known as the ‘five A’s’, namely, formulating an answerable clinical question (Asking), finding the best available answer to this question (Acquiring), critically evaluating the evidence (Appraising), applying the evidence (Applying) and monitoring the performance in relationship to the evidence (Assessing) (Spek et al. 2013). A scaffolded approach in which the steps of EBP are taught in different years of training has been reported to be effective: in the first-year online navigation, formulation of learning questions and referencing are taught, followed in the second year by formulation of PICO format questions, and searching for evidence using appropriate databases and by critically appraising the evidence. At the senior level, students learn how to develop evidence-based clinical guidelines, critical appraisal, research methodology and rating of evidence (Finotto et al. 2013; Spek et al. 2013).

## Problem statement

Insufficient attention has been paid to the use and teaching of EBP in nursing education, especially in middle- and low-income countries (Horntvedt et al. 2018; Hung et al. 2015). Much of the research on EBP has been undertaken in high-income countries. At the University of Rwanda, nursing students are expected to critically analyse current scientific evidence and apply EBP in their clinical practice; however, they have a limited 3-h exposure to EBP in the second year of the four-year programme, which does not include any practical literature searching skills and clear links to clinical practice. It is not known whether the integration of EBP in this programme is perceived by students to be useful in its application in a low-income country. A number of barriers to the application of EBP have been described in the literature (Lai, Teng & Lee 2010; Malik, Mckenna & Plummer 2016; McInerney & Suleman 2010), but the barriers specific to this setting have not been investigated.

This study aimed to determine the knowledge of, attitudes towards and application of EBP by third- and fourth-year nursing students.

## Methods

### Design

A cross-sectional survey design was utilised, using a self-reported questionnaire.

### Setting and population

The study was conducted at the School of Nursing and Midwifery, University of Rwanda, which is the largest nursing school in Rwanda. It was the first nursing school in the country to have an undergraduate degree programme in nursing. The target population was nursing students (*n* = 85) in the third and fourth years (48 in the third year; 37 in the fourth year). This group was selected as they met the following inclusion criteria:

- Students who had completed an EBP module at the School of Nursing (offered in the second year of the programme). This group, having been exposed to the module, would have been expected to apply EBP principles in their clinical placements.
- Informed, voluntary willingness to participate.

Students who had been transferred from other nursing schools into the third and fourth years of the programme were excluded, as it could not be established if their exposure to EBP was similar to that taught in the school of nursing at which the study was conducted.

### Data collection instrument

A knowledge, attitudes and behaviour (KAB) questionnaire to assess EBP in undergraduate students was used with permission (Johnston et al. 2003). This 43-item questionnaire has four subscales with questions that address the following: EBP knowledge; attitudes towards EBP; personal application and actual use of EBP, including self-perceived barriers to application; and anticipated future use of EBP. A Likert scale is used for each subscale, each with different response options, such as strongly agree to strongly disagree, every day to never or completely to not at all (Johnston et al. 2003). Validation of the questionnaire showed high construct validity: Cronbach’s alpha >0.7 for each scale and reliability Cronbach’s alpha of 0.71–0.88 (Johnston et al. 2003). Only the year of study of the student respondent was noted on the questionnaire; no other demographic data were collected.

### Data collection procedures

Arrangements were made with the head of the nursing school for a suitable date and time, which would not compromise on teaching time. Data were collected on the final day of the term after examinations had been completed. The school administration and lecturers were informed about the study but were not present during data collection. Questionnaires, each number-coded, were hand-distributed by the researcher to all respondents. All individually completed questionnaires were collected on the day of distribution.

### Ethical considerations

The principles of the Declaration of Helsinki were adhered to (World Medical Association 2013). Written informed consent was obtained from those respondents who agreed to participate. Students were free to voluntarily participate or withdraw without penalty. Questionnaires were coded and anonymous. All data were safely stored. The study was approved by the Human Research Ethics Committee at the Faculty of Health Sciences, the University of Cape Town (UCT) and the Directorate of Science, Technology (HREC: REF 253/2015) and Research at the Rwandan Ministry of Education (MINEDUC/S&T 305/2015). After ethics approval had been obtained, invitations to participate in the study, with information relevant to the study and dates for data collection, were distributed to third- and fourth-year students through their class representatives.

### Data analysis

Data were analysed using SPSS version 22. Univariate statistical analyses were performed to explore the distribution of data. Scoring was done according the recommendations of the authors (Johnston et al. 2003). This included the analysis of the negatively worded questions. The relationship between knowledge, attitudes and application of EBP with the year of study was examined using the *χ*^2^ tests and the significance level was set at *α* = 0.05 and the confidence interval at 95%.

## Results

All fourth-year students (*n* = 37) and 45 of the 48 third-year students completed the questionnaire, giving a response rate of 96%.

### Self-reported knowledge of evidence-based practice

Most students self-reported adequate knowledge of the five steps of the EBP process, with a range of 84% – 92% (Table 1). Seventy-five students (92%) agreed that effective searching and having easy access to evidence sources are essential for the application of EBP. The majority (90%) reported that they had a clear understanding of EBP and that EBP requires the identification and formulation of clinical questions. Sixty-nine students (84%) reported that research using clinical trials is more reliable than observational methods.

**TABLE 1:** Knowledge of evidence-based practice.

Knowledge-related variables	Disagree		Agree		*χ*^2^	df	*p*
	*n*	%	*n*	%			
I have a clear understanding of EBP.	9	10	73	90	6.703†	5	0.244
Research using clinical trials is generally more reliable than research using the observational method.	13	16	69	84	11.955†	5	0.035
The evidence-based practice process requires the appropriate identification and formulation of clinical questions.	8	10	74	90	5.482†	4	0.241
Effective searching/easy access to bibliographic databases and evidence sources are essential for evidence-based practice.	7	8	75	92	14.605†	5	0.012
Evidence-based practice requires the use of critical appraisal skills to ensure the quality of all research papers retrieved.	9	11	73	89	3.877†	4	0.423

† , *χ*^2^ statistically significant at *p* < 0.05.

EBP, evidence-based practice.

A *χ*^2^ test was run to evaluate the relationship between the knowledge of EBP and the year of study. A statistical significance was found between the year of study and two knowledge-related statements, ‘Research using clinical trials is more reliable than research using observation’ (*χ*^2^ = 11.955, *p* = 0.03) and ‘Effective searching skills/easy access to bibliographic databases and evidence sources are essential to practicing evidence-based practice’ (*χ*^2^ = 14.605, *p* = 0.01). More fourth-year than third-year students strongly agreed with both statements.

### Self-reported attitudes towards evidence-based practice

Most students scored positively on three attitude-related statements and negatively on two statements. Most students (63%) agreed that EBP is a cookbook or guidebook that disregards clinical experience; 56% agreed that if EBP is valid, then anyone can do what nurses do. Forty students (49%) agreed that previous work experience is more important than research findings. Sixty per cent of the students agreed that EBP is too time-consuming for busy nursing students. The only attitudinal variable that was associated with the year of study was the statement ‘EBP ignores the art of nursing’ (*χ*^2^ = 11.938, *p* = 0.03). More students from the fourth year disagreed with this statement than the third year. Almost all (97%) agreed that EBP should be an integral part of the undergraduate nursing curriculum (Table 2).

**TABLE 2:** Self-reported attitudes towards evidence-based practice.

Attitudes towards evidence-based practice	Disagree		Agree		*χ*^2^	df	*p*
	*n*	%	*n*	%			
Evidence-based practice is ‘cookbook’ (or ‘guidebook’) that disregards clinical experience.	30	37	52	63	9.894†	5	0.078
There is no reason for me personally to adopt EBP because it is just a ‘fad’ (or ‘fashion’) that will pass with time.	63	77	19	23	4.866†	5	0.432
If EBP is valid, then anyone can see patients and do what nurses do.	36	44	46	56	4.822†	5	0.438
EBP ignores the ‘art’ of nursing.	64	78	18	22	11.938†	5	0.036
Nurses, in general, should not practise EBP because nursing is about people and patients, not statistics.	61	74	21	26	7.877†	5	0.163
Previous work experience is more important than research findings in choosing the best treatment available for a patient.	42	51	40	49	9.418†	5	0.094

† , *χ*^2^ statistically significant at *p* < 0.05.

EBP, evidence-based practice; df, degrees of freedom.

### Self-reported application of evidence-based practice

Most students reported accessing evidence weekly or monthly, with fewer students (12%) reporting daily evidence searches. The main sources utilised were general online searches and textbooks. Databases such as Cochrane (1%), MEDLINE and CINAHL (6%) were rarely accessed. Student preferences for accessing evidence were a library (41%), a mobile/handheld computer (40%) and a computer in a patient care environment (39%).

The main barriers to the application of EBP identified were lack of knowledge (29%), lack of time (28%) and lack of examples or role modelling from lecturers, clinical instructors and nurses (21%). With reference to the future use of EBP, the majority of respondents (98%, *n* = 82) believed that EBP would be useful in their practice as nurses. There was a very positive response to EBP as the standard of care in clinical nursing, with 98% (*n* = 82) expressing a willingness to practise EBP (Table 3).

**TABLE 3:** Self-reported evidenced-based practice access.

Self-reported evidenced-based practice access	Never		Every month		Every week		Every other day		Every day		Total	
	*n*	%	*n*	%	*n*	%	*n*	%	*n*	%	*n*	%
How frequently do you access research evidence in general?	14	17	26	32	17	21	15	18	10	12	82	100
How frequently do you access research evidence via the Internet?	6	7	15	18	22	27	31	38	8	10	82	100
How frequently do you access research evidence from a textbook?	14	17	30	37	14	17	20	24	4	5	82	100
How frequently do you access research evidence from original research papers?	36	44	21	26	13	16	11	13	1	1	82	100
How frequently do you access research evidence from the Cochrane database?	59	72	8	10	4	5	9	11	2	2	82	100

### Self-reported barriers

Students reported lack of knowledge (29%), lack of time (28%) and lack of examples or role modelling from lecturers, clinical instructors and nurses (21%) as the main barriers to their application of EBP.

## Discussion

The study found that most third- and fourth-year nursing students at the school of nursing were knowledgeable about the five steps of EBP. Our findings are consistent with those reported by researchers in an Ireland-based study; in this study, most nursing students had a clear understanding of EBP, agreed that clinical trials were more reliable than observation methods and also agreed that effective searching skills were necessary for the effective application of EBP (Stronge & Cahill 2012). Knowledge of the EBP process is the initial step towards developing positive attitudes and applying EBP in clinical practice; however, theoretical knowledge alone may not trigger positive attitudes or increase application in practice.

An association between two of the EBP steps with the year of study was observed. These two steps require knowledge about research methodology; therefore, this association may be considered as a result of an increased exposure to research. Fourth-year students at the university complete a minor research project that entails reviewing literature, collecting data and reporting. Nurses who are exposed to a research course with an application component are more likely to have more knowledge and positive attitudes towards EBP, compared with those without exposure (Melnyk et al. 2012). Research courses alone, however, may not provide students with sufficient background to develop EBP skills. Nurses may struggle with evaluating and applying research findings in spite of the traditional research training (Finotto et al. 2013).

Three of the six attitudinal variables scored positively, and two variables scored negatively, with no significant differences between the attitudes of the third- and fourth-year students. Similar results were reported in a Saudi Arabian study involving final-year dental and medical students (Bahammam & Linjawi 2014). Stronge and Cahill (2012) in their Ireland-based study, however, reported positive attitudes towards EBP. Attitudes towards EBP are shaped by knowledge and skills; therefore, for most persons, adequate knowledge and skills would be associated with positive attitudes. Theoretical knowledge alone is not enough to shape positive attitudes, which may explain the disconnect between knowledge and attitudes observed in the present study. Stokke et al. (2014) reported that nurses who have knowledge and competence in EBP, access to resources and experience support have more belief in EBP. The translation of knowledge into practice through a continuous and guided EBP learning process in both the clinical and classroom settings (Finotto et al. 2013) is essential. Effective role models and mentors are integral to the forming of student attitudes and behaviour in practice (Ryan 2016).

Almost all students in this study agreed that EBP should be an integral part of the nursing curriculum. Integration of EBP in health professional curricula across different levels with related assessment methods has been recommended in a number of studies (Alving, Christensen & Thrysøe 2018; Melnyk et al. 2012, 2014). In an integrative review, Ryan (2016) argued that research and EBP should be incorporated into the undergraduate nursing curriculum and integrated into the clinical environment.

Students reported limited access to and application of EBP compared to what is reported by students in the Western countries, and they used the Internet and textbooks as their main sources of evidence. Reliable databases or search engines were rarely used. Similar findings have been reported in other studies (Brown et al. 2010; Reid et al. 2017). In this study, textbooks and the general searches online were the primary sources of evidence for nursing students before receiving the intervention. This may transition into the work setting, as there is evidence that nurses in clinical settings are less likely to make use of reliable databases, such as Cochrane, PubMed and CINAHL, preferring instead to consult peers or utilise general search engines as their preferred sources of evidence (Alving et al. 2018).

Students in this study reported lack of support and example from lecturers, clinical instructors and qualified nurses as a barrier to the application of EBP. The paucity of role models has been reported as a barrier to the application of EBP (Jonsén, Melender & Hilli 2013; Tacia et al. 2015). Traditional didactic teaching methods should be replaced with innovative teaching strategies that increase students’ EBP knowledge and skills (Kim et al. 2009), and offer a unique opportunity to influence the nursing students’ perceptions and practice with regard to EBP, which, in turn, impacts current and future practice of EBP. Lack of knowledge and time reported in this study is consistent with the findings in other studies (Malik et al. 2016; McInerney & Suleman 2010). There is a contrast between students reporting adequate knowledge of the EBP process and lack of knowledge as a barrier to the application of EBP, which may be because of lack of skills or practical knowledge to apply EBP. Lack of knowledge and limited access to user-friendly technology inhibit the application of EBP, which is also influenced by a limited ability to use computers or the available software (Tacia et al. 2015).

## Limitations of the study

The study was limited to a university nursing school in Rwanda; therefore, the results cannot be generalised to all nursing students. As in the original study conducted by Johnston et al. (2003), the only demographic variable collected was that of year of study. More demographic data would have allowed us to test for more relationships. As a cross-sectional study, no cause–effect relationship can be assumed. In addition, self-assessment studies may show an overestimation of the respondents’ level of knowledge.

## Areas of future research

This study did not investigate the EBP knowledge and skills of educators and clinical instructors at the school of nursing, which may influence the learning opportunities for students. Studies that investigate the application of EBP learning to practice should be conducted.

## Conclusion

The role of nurse educators and clinical instructors in teaching EBP is critical: lack of support from clinical instructors may constitute a very large barrier to the application of EBP. Practice change requires theoretical input, the development of related skills, practical application and effective role models. To facilitate the development of competent nurses who use evidence to monitor and enhance patient care, EBP teaching at the school of nursing needs to be an integral part of the nursing curriculum throughout the undergraduate programme, with emphasis on both theory and practice.
